# 8-(Biphenyl-2-yl)-7,9-diphenyl-8*H*-cyclo­penta­[*a*]acenaphthylen-8-ol

**DOI:** 10.1107/S1600536809011222

**Published:** 2009-03-31

**Authors:** Peter G. Jones, Marc Debeaux, Henning Hopf, Wolfgang Kowalsky, Hans-Hermann Johannes

**Affiliations:** a Institut für Anorganische und Analytische Chemie, Technical University of Braunschweig, Postfach 3329, 38023 Braunschweig, Germany; b Labor für Elektro­optik am Institut für Hochfrequenztechnik, Technical University of Braunschweig, Postfach 3329, 38023 Braunschweig, Germany; c Institut für Organische Chemie, Technical University of Braunschweig, Postfach 3329, 38023 Braunschweig, Germany

## Abstract

In the title compound, C_39_H_26_O, the cyclo­penta­[*a*]acenaphthyl­ene skeleton displays the expected distortions, with formal *sp* ^2^ bond angles as high as C—C—C = 142.50 (10)°. The OH group forms inter­molecular hydrogen bonds *via x*-axis translation to the centroid (*Cg*) of the pendant phenyl ring of the biphenyl system, with H⋯*Cg* = 2.41 Å and O—H⋯*Cg* = 153°.

## Related literature

For related literature, see: Saragi *et al.* (2007 ▶); Velusamy *et al.* (2007 ▶).
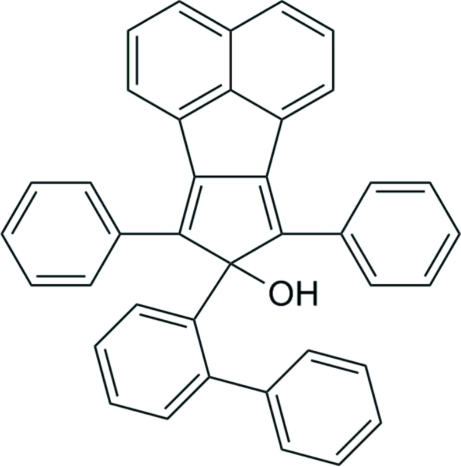


## Experimental

### 

#### Crystal data

C_39_H_26_O
*M* *_r_* = 510.60
Monoclinic, 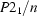

*a* = 7.3837 (4) Å
*b* = 18.4001 (12) Å
*c* = 19.2505 (12) Å
β = 97.549 (3)°
*V* = 2592.7 (3) Å^3^
*Z* = 4
Mo *K*α radiation
μ = 0.08 mm^−1^
*T* = 103 K
0.35 × 0.20 × 0.20 mm

#### Data collection

Bruker APEXII CCD area-detector diffractometer
Absorption correction: multi-scan (*SADABS*; Bruker, 2004 ▶) *T*_min_ = 0.893, *T*_max_ = 0.985
53233 measured reflections
7857 independent reflections
6335 reflections with *I* > 2σ(*I*)
*R*_int_ = 0.036

#### Refinement

*R*[*F* ^2^ > 2σ(*F* ^2^)] = 0.044
*wR*(*F* ^2^) = 0.125
*S* = 1.02
7857 reflections
365 parameters
H atoms treated by a mixture of independent and constrained refinement
Δρ_max_ = 0.46 e Å^−3^
Δρ_min_ = −0.22 e Å^−3^

### 

Data collection: *APEX2* (Bruker, 2004 ▶); cell refinement: *SAINT* (Bruker, 2004 ▶); data reduction: *SAINT*; program(s) used to solve structure: *SHELXS97* (Sheldrick, 2008 ▶); program(s) used to refine structure: *SHELXL97* (Sheldrick, 2008 ▶); molecular graphics: *XP* (Siemens, 1994 ▶); software used to prepare material for publication: *SHELXL97*.

## Supplementary Material

Crystal structure: contains datablocks I, global. DOI: 10.1107/S1600536809011222/bt2916sup1.cif

Structure factors: contains datablocks I. DOI: 10.1107/S1600536809011222/bt2916Isup2.hkl

Additional supplementary materials:  crystallographic information; 3D view; checkCIF report

## Figures and Tables

**Fig. 1.**
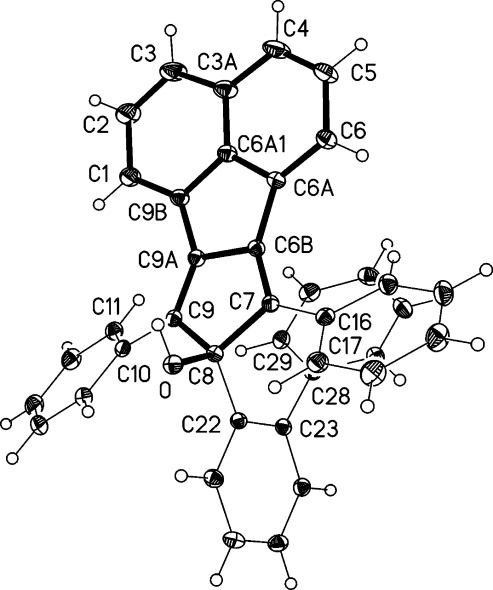
The formula unit of the title compound in the crystal. Ellipsoids represent 50% probability levels. Only the first two atoms of phenyl rings are numbered; other atoms follow in sequence.

**Fig. 2.**
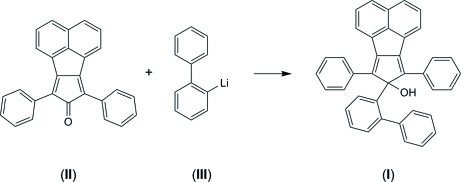
Preparation of the title compound.

## supplementary crystallographic information

### Comment

The title compound (I) is a derivative of 7,9-diphenyl-8*H*-cyclopenta[*a*]acenaphthylen-8-one, an interesting starting compound for optoelectronic materials (Velusamy *et al.*, 2007). Spiro compounds with orthogonally fixed aromatic moieties are known to form stable molecular glasses, an important prerequisite of materials for optoelectronic applications (Saragi *et al.*, 2007). We have synthesized the title compound by addition of biphenyl-2-yl lithium (III) to the ketone II with a view to generating a corresponding spiro compound, which could combine both attractive electronic properties and amorphous stability, by further cyclocondensation.

The molecule of I is shown in Fig. 1. The cyclopenta[*a*]acenaphthylene skeleton displays the expected distortions, with formally *sp*^2^ angles as high as C7—C6B—C6A 142.50 (10)°. The 15 atoms of this skeleton are reasonably coplanar (r.m.s.d. 0.09 Å) but a better description is of the fused cyclopentadiene (r.m.s.d. 0.030 Å) subtending an interplanar angle of 10.52 (1)° with the ten atoms of the naphthalenoid moiety plus the atoms C6B and C9A (r.m.s.d. 0.037 Å). With the phenyl rings C10–15, C16–21, C22–27 it subtends angles of 33.50 (4), 30.51 (4) and 83.89 (4)° respectively. The biphenyl interplanar angle is 76.93 (4)°.

The OH group does not form intermolecular hydrogen bonds with its counterparts in neighbouring molecules, presumably for steric reasons. Instead, the acceptor is the centroid of ring C28–33, with H01···Cent 2.41 Å, O—H01···Cent 153°, operator *x* - 1,*y*,*z*.

### Experimental

2-Bromobiphenyl (900 mg, 3.86 mmol) in dry THF (10 ml) was treated with a 1.6 *M* solution of *n*-butyl lithium in *n*-hexane (2.81 ml, 4.50 mmol) at -80 °C. The mixture was stirred for 1 h at -80 °C and added to a suspension of 7,9-diphenyl-8*H*-cyclopenta[*a*]acenaphthylen-8-one (1.38 g, 3.86 mmol) in dry THF (25 ml). After 4 h of stirring under reflux, a saturated aqueous solution of ammonium chloride (50 ml) was added. Extraction with dichloromethane (3 × 50 ml), drying (MgSO_4_) and concentration afforded the crude product, which was purified by flash chromatography on silica gel (CH_2_Cl_2_/*n*-hexane, 1:1; *R*_f _= 0.32). The title compound was obtained as a yellow microcrystalline solid (597 mg, 30%), mp 244 °C. Elemental analysis: calculated for C_39_H_26_O: C 91.73, H 5.13%; found: C 91.79, 5.07%. Spectroscopic analysis: ^1^H NMR (400 MHz, CDCl_3_) δ = 8.29 (dd, J = 8.1, 1.3, 1 H), 7.75 (d, J = 7.1, 2 H), 7.68–7.62 (m, 6 H), 7.48–7.39 (m, 3 H), 7.37–7.24 (m, 7 H), 6.92 (dd, J = 7.5, 1.4, 1 H), 6.83 (dd, J = 7.9, 1.5, 2 H), 6.47 (dd, J = 7.7, 7.7, 2 H), 6.27–6.21 (m, 1 H), 2.50 p.p.m. (s, 1 H, OH); ^13^C NMR (101 MHz, CDCl_3_) δ = 145.2 (*s*), 144.1 (*s*), 141.0 (*s*), 140.5 (*s*), 140.4 (*s*), 137.1 (*s*), 134.4 (*s*), 132.4 (*s*), 131.6 (*s*), 131.4 (*d*), 128.8 (*d*), 128.2 (*d*), 128.1 (*d*), 127.7 (*d*), 127.5 (*d*), 127.4 (*d*), 127.4 (*d*), 127.1 (*d*), 126.0 (*d*), 125.6 (*d*), 125.5 (*d*), 119.2 (*d*), 96.3 p.p.m. (*s*).

### Refinement

The hydroxyl hydrogen was identified in a difference synthesis and refined freely. Other hydrogen atoms were included using a riding model with C—H 0.95 Å; U(H) values were fixed at 1.2 × *U*_eq_(C) of the parent C atom.

### Crystal data

**Table:**

C_39_H_26_O	*F*(000) = 1072
*M**_r_* = 510.60	*D*_x_ = 1.308 Mg m^−^^3^
Monoclinic, *P*2_1_/*n*	Mo *K*α radiation, λ = 0.71073 Å
*a* = 7.3837 (4) Å	Cell parameters from 9892 reflections
*b* = 18.4001 (12) Å	θ = 2.4–31.4°
*c* = 19.2505 (12) Å	µ = 0.08 mm^−^^1^
β = 97.549 (3)°	*T* = 103 K
*V* = 2592.7 (3) Å^3^	Prism, yellow
*Z* = 4	0.35 × 0.20 × 0.20 mm

### Data collection

**Table:**

Bruker APEXII CCD area-detector diffractometer	7857 independent reflections
Radiation source: fine-focus sealed tube	6335 reflections with *I* > 2σ(*I*)
graphite	*R*_int_ = 0.036
φ and ω scans	θ_max_ = 30.5°, θ_min_ = 2.4°
Absorption correction: multi-scan (*SADABS*; Bruker, 2004)	*h* = −10→9
*T*_min_ = 0.893, *T*_max_ = 0.985	*k* = −26→26
53233 measured reflections	*l* = −27→27

### Refinement

**Table:**

Refinement on *F*^2^	Primary atom site location: structure-invariant direct methods
Least-squares matrix: full	Secondary atom site location: difference Fourier map
*R*[*F*^2^ > 2σ(*F*^2^)] = 0.044	Hydrogen site location: inferred from neighbouring sites
*wR*(*F*^2^) = 0.125	H atoms treated by a mixture of independent and constrained refinement
*S* = 1.02	*w* = 1/[σ^2^(*F*_o_^2^) + (0.0699*P*)^2^ + 0.9032*P*] where *P* = (*F*_o_^2^ + 2*F*_c_^2^)/3
7857 reflections	(Δ/σ)_max_ = 0.001
365 parameters	Δρ_max_ = 0.46 e Å^−^^3^
0 restraints	Δρ_min_ = −0.22 e Å^−^^3^

### Special details

**Table:**

Geometry. All e.s.d.'s (except the e.s.d. in the dihedral angle between two l.s. planes) are estimated using the full covariance matrix. The cell e.s.d.'s are taken into account individually in the estimation of e.s.d.'s in distances, angles and torsion angles; correlations between e.s.d.'s in cell parameters are only used when they are defined by crystal symmetry. An approximate (isotropic) treatment of cell e.s.d.'s is used for estimating e.s.d.'s involving l.s. planes.Least-squares planes (*x*,*y*,*z* in crystal coordinates) and deviations from them (* indicates atom used to define plane)7.3583 (0.0005) *x* + 0.7119 (0.0054) *y* - 3.9201 (0.0047) *z* = 0.2623 (0.0032)* -0.0009 (0.0010) C1 * -0.0525 (0.0012) C2 * -0.0232 (0.0012) C3 * 0.0267 (0.0012) C3A * 0.0072 (0.0012) C4 * -0.0118 (0.0011) C5 * -0.0169 (0.0010) C6 * 0.0149 (0.0009) C6A * 0.0575 (0.0010) C6A1 * -0.0612 (0.0008) C6B * 0.0602 (0.0010) C9BRms deviation of fitted atoms = 0.03747.3441 (0.0006) *x* - 1.4269 (0.0094) *y* - 1.2091 (0.0098) *z* = 1.2752 (0.0064)Angle to previous plane (with approximate e.s.d.) = 10.52 (0.01)* 0.0010 (0.0006) C6B * -0.0289 (0.0006) C9A * 0.0416 (0.0006) C9 * -0.0370 (0.0006) C8 * 0.0233 (0.0006) C7Rms deviation of fitted atoms = 0.02995.9686 (0.0021) *x* - 10.0295 (0.0075) *y* + 2.1998 (0.0091) *z* = 1.0584 (0.0079)Angle to previous plane (with approximate e.s.d.) = 30.51 (0.04)* -0.0149 (0.0007) C16 * 0.0092 (0.0008) C17 * 0.0028 (0.0009) C18 * -0.0090 (0.0009) C19 * 0.0030 (0.0008) C20 * 0.0090 (0.0008) C21Rms deviation of fitted atoms = 0.0090===========================================================================5.7246 (0.0022) *x* - 6.2243 (0.0078) *y* + 8.2177 (0.0079) *z* = 5.2956 (0.0036)Angle to previous plane (with approximate e.s.d.) = 21.61 (0.05)* 0.0022 (0.0007) C10 * -0.0022 (0.0008) C11 * -0.0003 (0.0008) C12 * 0.0027 (0.0008) C13 * -0.0026 (0.0008) C14 * 0.0002 (0.0008) C15Rms deviation of fitted atoms = 0.00207.3441 (0.0006) *x* - 1.4269 (0.0094) *y* - 1.2091 (0.0098) *z* = 1.2752 (0.0064)Angle to previous plane (with approximate e.s.d.) = 33.50 (0.04)* 0.0010 (0.0006) C6B * -0.0289 (0.0006) C9A * 0.0416 (0.0006) C9 * -0.0370 (0.0006) C8 * 0.0233 (0.0006) C7Rms deviation of fitted atoms = 0.0299- 1.0281 (0.0031) *x* + 7.1737 (0.0070) *y* + 17.7236 (0.0033) *z* = 12.7054 (0.0026)Angle to previous plane (with approximate e.s.d.) = 83.89 (0.04)* -0.0019 (0.0007) C22 * 0.0011 (0.0007) C23 * 0.0018 (0.0007) C24 * -0.0038 (0.0007) C25 * 0.0030 (0.0007) C26 * -0.0001 (0.0007) C27Rms deviation of fitted atoms = 0.00236.5435 (0.0016) *x* - 8.3888 (0.0073) *y* - 0.6702 (0.0088) *z* = 2.5647 (0.0063)Angle to previous plane (with approximate e.s.d.) = 76.93 (0.04)* -0.0148 (0.0007) C28 * 0.0087 (0.0007) C29 * 0.0045 (0.0008) C30 * -0.0117 (0.0008) C31 * 0.0055 (0.0008) C32 * 0.0078 (0.0008) C33Rms deviation of fitted atoms = 0.0095
Refinement. Refinement of *F*^2^ against ALL reflections. The weighted *R*-factor *wR* and goodness of fit *S* are based on *F*^2^, conventional *R*-factors *R* are based on *F*, with *F* set to zero for negative *F*^2^. The threshold expression of *F*^2^ > σ(*F*^2^) is used only for calculating *R*-factors(gt) *etc*. and is not relevant to the choice of reflections for refinement. *R*-factors based on *F*^2^ are statistically about twice as large as those based on *F*, and *R*- factors based on ALL data will be even larger.

### Fractional atomic coordinates and isotropic or equivalent isotropic displacement parameters (Å^2^)

**Table:**

	*x*	*y*	*z*	*U*_iso_*/*U*_eq_	
O	0.13493 (10)	0.15448 (4)	0.67183 (4)	0.01423 (15)	
H01	0.053 (3)	0.1864 (10)	0.6540 (9)	0.036 (5)*	
C1	0.24115 (17)	0.31692 (6)	0.44354 (6)	0.0200 (2)	
H1	0.2319	0.2716	0.4197	0.024*	
C2	0.2079 (2)	0.38281 (7)	0.40623 (6)	0.0265 (3)	
H2	0.1741	0.3809	0.3569	0.032*	
C3	0.2226 (2)	0.44958 (7)	0.43856 (7)	0.0282 (3)	
H3	0.2022	0.4926	0.4113	0.034*	
C3A	0.26827 (18)	0.45465 (6)	0.51240 (6)	0.0211 (2)	
C4	0.2812 (2)	0.51906 (6)	0.55328 (7)	0.0267 (3)	
H4	0.2648	0.5651	0.5309	0.032*	
C5	0.31712 (18)	0.51523 (6)	0.62491 (7)	0.0233 (2)	
H5	0.3255	0.5591	0.6511	0.028*	
C6	0.34231 (15)	0.44817 (6)	0.66134 (6)	0.0168 (2)	
H6	0.3655	0.4473	0.7111	0.020*	
C6A	0.33272 (14)	0.38416 (5)	0.62358 (5)	0.01327 (19)	
C6B	0.33853 (14)	0.30597 (5)	0.63970 (5)	0.01192 (18)	
C6A1	0.29829 (15)	0.38892 (6)	0.54897 (5)	0.0151 (2)	
C7	0.34110 (14)	0.25807 (5)	0.69336 (5)	0.01173 (18)	
C8	0.31266 (13)	0.18120 (5)	0.66124 (5)	0.01113 (18)	
C9	0.31279 (14)	0.19389 (5)	0.58200 (5)	0.01183 (18)	
C9A	0.31558 (14)	0.26635 (5)	0.57176 (5)	0.01246 (18)	
C9B	0.28743 (15)	0.31995 (6)	0.51537 (5)	0.01469 (19)	
C10	0.31073 (14)	0.13514 (6)	0.53058 (5)	0.01317 (19)	
C11	0.40373 (15)	0.14418 (6)	0.47211 (5)	0.0160 (2)	
H11	0.4617	0.1892	0.4653	0.019*	
C12	0.41254 (16)	0.08846 (7)	0.42400 (6)	0.0201 (2)	
H12	0.4764	0.0956	0.3848	0.024*	
C13	0.32818 (17)	0.02248 (7)	0.43315 (6)	0.0217 (2)	
H13	0.3346	−0.0158	0.4004	0.026*	
C14	0.23426 (16)	0.01256 (6)	0.49042 (6)	0.0197 (2)	
H14	0.1755	−0.0325	0.4965	0.024*	
C15	0.22550 (15)	0.06821 (6)	0.53901 (6)	0.0164 (2)	
H15	0.1614	0.0607	0.5781	0.020*	
C16	0.34884 (14)	0.27215 (6)	0.76866 (5)	0.01294 (19)	
C17	0.26048 (16)	0.22669 (6)	0.81206 (6)	0.0170 (2)	
H17	0.1996	0.1842	0.7931	0.020*	
C18	0.26098 (17)	0.24311 (7)	0.88270 (6)	0.0218 (2)	
H18	0.1990	0.2121	0.9113	0.026*	
C19	0.35097 (18)	0.30421 (7)	0.91169 (6)	0.0230 (2)	
H19	0.3495	0.3155	0.9598	0.028*	
C20	0.44350 (16)	0.34891 (6)	0.86992 (6)	0.0201 (2)	
H20	0.5069	0.3905	0.8896	0.024*	
C21	0.44323 (15)	0.33267 (6)	0.79935 (6)	0.0158 (2)	
H21	0.5082	0.3632	0.7714	0.019*	
C22	0.45086 (14)	0.12448 (5)	0.69252 (5)	0.01134 (18)	
C23	0.64062 (14)	0.13629 (5)	0.69892 (5)	0.01170 (18)	
C24	0.75989 (14)	0.08196 (6)	0.72787 (5)	0.01409 (19)	
H24	0.8879	0.0901	0.7323	0.017*	
C25	0.69485 (15)	0.01641 (6)	0.75031 (6)	0.0156 (2)	
H25	0.7775	−0.0202	0.7694	0.019*	
C26	0.50835 (15)	0.00505 (6)	0.74447 (6)	0.0159 (2)	
H26	0.4624	−0.0394	0.7602	0.019*	
C27	0.38801 (14)	0.05834 (6)	0.71575 (5)	0.01441 (19)	
H27	0.2602	0.0496	0.7118	0.017*	
C28	0.72295 (13)	0.20575 (5)	0.67853 (5)	0.01229 (18)	
C29	0.74001 (14)	0.22183 (6)	0.60887 (6)	0.0151 (2)	
H29	0.6963	0.1884	0.5729	0.018*	
C30	0.82100 (15)	0.28686 (6)	0.59174 (6)	0.0185 (2)	
H30	0.8314	0.2978	0.5442	0.022*	
C31	0.88645 (15)	0.33565 (6)	0.64425 (6)	0.0198 (2)	
H31	0.9391	0.3804	0.6326	0.024*	
C32	0.87449 (16)	0.31872 (6)	0.71380 (6)	0.0190 (2)	
H32	0.9217	0.3515	0.7498	0.023*	
C33	0.79400 (15)	0.25429 (6)	0.73091 (6)	0.0153 (2)	
H33	0.7871	0.2430	0.7787	0.018*	

### Atomic displacement parameters (Å^2^)

**Table:**

	*U*^11^	*U*^22^	*U*^33^	*U*^12^	*U*^13^	*U*^23^
O	0.0097 (3)	0.0152 (3)	0.0180 (4)	−0.0002 (3)	0.0025 (3)	0.0021 (3)
C1	0.0290 (6)	0.0174 (5)	0.0134 (5)	0.0004 (4)	0.0021 (4)	0.0017 (4)
C2	0.0419 (8)	0.0229 (6)	0.0145 (5)	0.0025 (5)	0.0021 (5)	0.0054 (4)
C3	0.0460 (8)	0.0190 (6)	0.0194 (6)	0.0038 (5)	0.0039 (5)	0.0081 (4)
C3A	0.0292 (6)	0.0155 (5)	0.0193 (5)	0.0018 (4)	0.0058 (4)	0.0040 (4)
C4	0.0419 (8)	0.0121 (5)	0.0268 (6)	0.0029 (5)	0.0073 (5)	0.0039 (4)
C5	0.0329 (6)	0.0123 (5)	0.0256 (6)	0.0011 (4)	0.0065 (5)	−0.0016 (4)
C6	0.0192 (5)	0.0139 (5)	0.0178 (5)	0.0007 (4)	0.0042 (4)	−0.0016 (4)
C6A	0.0133 (4)	0.0114 (4)	0.0154 (4)	0.0001 (3)	0.0030 (3)	0.0003 (3)
C6B	0.0118 (4)	0.0114 (4)	0.0127 (4)	0.0008 (3)	0.0023 (3)	−0.0009 (3)
C6A1	0.0172 (5)	0.0129 (5)	0.0154 (5)	0.0003 (4)	0.0032 (4)	0.0016 (4)
C7	0.0108 (4)	0.0119 (4)	0.0125 (4)	0.0011 (3)	0.0018 (3)	−0.0011 (3)
C8	0.0107 (4)	0.0112 (4)	0.0117 (4)	0.0001 (3)	0.0022 (3)	0.0000 (3)
C9	0.0116 (4)	0.0127 (4)	0.0110 (4)	0.0003 (3)	0.0010 (3)	−0.0005 (3)
C9A	0.0133 (4)	0.0123 (4)	0.0120 (4)	0.0001 (3)	0.0024 (3)	−0.0004 (3)
C9B	0.0173 (5)	0.0129 (4)	0.0141 (5)	0.0002 (4)	0.0029 (4)	0.0013 (4)
C10	0.0134 (4)	0.0131 (4)	0.0122 (4)	0.0014 (3)	−0.0013 (3)	−0.0015 (3)
C11	0.0173 (5)	0.0162 (5)	0.0141 (4)	0.0014 (4)	0.0011 (4)	−0.0015 (4)
C12	0.0226 (5)	0.0234 (6)	0.0139 (5)	0.0043 (4)	0.0015 (4)	−0.0040 (4)
C13	0.0257 (6)	0.0197 (5)	0.0182 (5)	0.0051 (4)	−0.0031 (4)	−0.0073 (4)
C14	0.0222 (5)	0.0138 (5)	0.0215 (5)	−0.0002 (4)	−0.0036 (4)	−0.0039 (4)
C15	0.0172 (5)	0.0153 (5)	0.0160 (5)	−0.0009 (4)	−0.0002 (4)	−0.0013 (4)
C16	0.0130 (4)	0.0132 (4)	0.0127 (4)	0.0033 (3)	0.0020 (3)	−0.0003 (3)
C17	0.0202 (5)	0.0164 (5)	0.0145 (5)	−0.0002 (4)	0.0031 (4)	0.0011 (4)
C18	0.0265 (6)	0.0255 (6)	0.0146 (5)	−0.0008 (5)	0.0067 (4)	0.0016 (4)
C19	0.0272 (6)	0.0286 (6)	0.0135 (5)	0.0018 (5)	0.0044 (4)	−0.0039 (4)
C20	0.0215 (5)	0.0213 (5)	0.0169 (5)	−0.0004 (4)	0.0007 (4)	−0.0058 (4)
C21	0.0163 (5)	0.0161 (5)	0.0149 (5)	0.0001 (4)	0.0019 (4)	−0.0018 (4)
C22	0.0127 (4)	0.0109 (4)	0.0106 (4)	0.0008 (3)	0.0019 (3)	−0.0006 (3)
C23	0.0134 (4)	0.0114 (4)	0.0105 (4)	0.0001 (3)	0.0022 (3)	−0.0010 (3)
C24	0.0129 (4)	0.0148 (5)	0.0144 (4)	0.0010 (4)	0.0011 (3)	−0.0010 (4)
C25	0.0181 (5)	0.0136 (5)	0.0151 (5)	0.0039 (4)	0.0021 (4)	0.0010 (4)
C26	0.0195 (5)	0.0112 (4)	0.0178 (5)	0.0007 (4)	0.0051 (4)	0.0020 (4)
C27	0.0138 (5)	0.0133 (4)	0.0165 (5)	−0.0010 (4)	0.0035 (4)	0.0008 (4)
C28	0.0096 (4)	0.0118 (4)	0.0157 (4)	0.0008 (3)	0.0021 (3)	0.0008 (3)
C29	0.0133 (5)	0.0169 (5)	0.0147 (5)	−0.0003 (4)	0.0011 (4)	0.0003 (4)
C30	0.0160 (5)	0.0204 (5)	0.0197 (5)	−0.0007 (4)	0.0038 (4)	0.0054 (4)
C31	0.0154 (5)	0.0153 (5)	0.0291 (6)	−0.0018 (4)	0.0042 (4)	0.0037 (4)
C32	0.0172 (5)	0.0149 (5)	0.0249 (5)	−0.0022 (4)	0.0025 (4)	−0.0035 (4)
C33	0.0148 (5)	0.0147 (5)	0.0163 (5)	−0.0005 (4)	0.0021 (4)	−0.0014 (4)

### Geometric parameters (Å, °)

**Table:**

O—C8	1.4408 (12)	C14—C15	1.3940 (15)
O—H01	0.879 (19)	C14—H14	0.9500
C1—C9B	1.3813 (15)	C15—H15	0.9500
C1—C2	1.4140 (16)	C16—C17	1.4024 (15)
C1—H1	0.9500	C16—C21	1.4024 (15)
C2—C3	1.3750 (18)	C17—C18	1.3925 (15)
C2—H2	0.9500	C17—H17	0.9500
C3—C3A	1.4201 (17)	C18—C19	1.3855 (18)
C3—H3	0.9500	C18—H18	0.9500
C3A—C6A1	1.4023 (15)	C19—C20	1.3908 (17)
C3A—C4	1.4188 (17)	C19—H19	0.9500
C4—C5	1.3714 (18)	C20—C21	1.3907 (15)
C4—H4	0.9500	C20—H20	0.9500
C5—C6	1.4194 (16)	C21—H21	0.9500
C5—H5	0.9500	C22—C27	1.3967 (14)
C6—C6A	1.3810 (14)	C22—C23	1.4073 (14)
C6—H6	0.9500	C23—C24	1.3988 (14)
C6A—C6A1	1.4280 (14)	C23—C28	1.4898 (14)
C6A—C6B	1.4711 (14)	C24—C25	1.3879 (15)
C6B—C7	1.3561 (14)	C24—H24	0.9500
C6B—C9A	1.4876 (14)	C25—C26	1.3827 (15)
C6A1—C9B	1.4219 (14)	C25—H25	0.9500
C7—C16	1.4663 (14)	C26—C27	1.3885 (14)
C7—C8	1.5470 (14)	C26—H26	0.9500
C8—C22	1.5269 (14)	C27—H27	0.9500
C8—C9	1.5433 (14)	C28—C29	1.3950 (14)
C9—C9A	1.3485 (14)	C28—C33	1.3967 (14)
C9—C10	1.4644 (14)	C29—C30	1.3964 (15)
C9A—C9B	1.4611 (14)	C29—H29	0.9500
C10—C15	1.4021 (15)	C30—C31	1.3903 (17)
C10—C11	1.4032 (15)	C30—H30	0.9500
C11—C12	1.3889 (15)	C31—C32	1.3886 (17)
C11—H11	0.9500	C31—H31	0.9500
C12—C13	1.3862 (18)	C32—C33	1.3852 (15)
C12—H12	0.9500	C32—H32	0.9500
C13—C14	1.3896 (18)	C33—H33	0.9500
C13—H13	0.9500		
			
C8—O—H01	107.9 (12)	C13—C14—C15	120.52 (11)
C9B—C1—C2	118.52 (11)	C13—C14—H14	119.7
C9B—C1—H1	120.7	C15—C14—H14	119.7
C2—C1—H1	120.7	C14—C15—C10	120.43 (10)
C3—C2—C1	122.55 (11)	C14—C15—H15	119.8
C3—C2—H2	118.7	C10—C15—H15	119.8
C1—C2—H2	118.7	C17—C16—C21	117.85 (10)
C2—C3—C3A	120.40 (11)	C17—C16—C7	121.52 (9)
C2—C3—H3	119.8	C21—C16—C7	120.63 (9)
C3A—C3—H3	119.8	C18—C17—C16	120.70 (10)
C6A1—C3A—C4	116.57 (10)	C18—C17—H17	119.7
C6A1—C3A—C3	116.54 (11)	C16—C17—H17	119.7
C4—C3A—C3	126.86 (11)	C19—C18—C17	120.60 (11)
C5—C4—C3A	120.32 (11)	C19—C18—H18	119.7
C5—C4—H4	119.8	C17—C18—H18	119.7
C3A—C4—H4	119.8	C18—C19—C20	119.54 (10)
C4—C5—C6	122.47 (11)	C18—C19—H19	120.2
C4—C5—H5	118.8	C20—C19—H19	120.2
C6—C5—H5	118.8	C21—C20—C19	119.99 (11)
C6A—C6—C5	119.14 (10)	C21—C20—H20	120.0
C6A—C6—H6	120.4	C19—C20—H20	120.0
C5—C6—H6	120.4	C20—C21—C16	121.26 (10)
C6—C6A—C6A1	117.85 (9)	C20—C21—H21	119.4
C6—C6A—C6B	136.45 (10)	C16—C21—H21	119.4
C6A1—C6A—C6B	105.59 (9)	C27—C22—C23	118.39 (9)
C7—C6B—C6A	142.50 (10)	C27—C22—C8	119.27 (9)
C7—C6B—C9A	109.86 (9)	C23—C22—C8	122.35 (9)
C6A—C6B—C9A	107.27 (8)	C24—C23—C22	119.47 (9)
C3A—C6A1—C9B	123.07 (10)	C24—C23—C28	117.43 (9)
C3A—C6A1—C6A	123.60 (10)	C22—C23—C28	123.06 (9)
C9B—C6A1—C6A	113.25 (9)	C25—C24—C23	121.30 (10)
C6B—C7—C16	129.27 (9)	C25—C24—H24	119.3
C6B—C7—C8	107.59 (8)	C23—C24—H24	119.3
C16—C7—C8	122.84 (9)	C26—C25—C24	119.20 (10)
O—C8—C22	106.55 (8)	C26—C25—H25	120.4
O—C8—C9	108.08 (8)	C24—C25—H25	120.4
C22—C8—C9	113.96 (8)	C25—C26—C27	120.26 (10)
O—C8—C7	109.50 (8)	C25—C26—H26	119.9
C22—C8—C7	114.75 (8)	C27—C26—H26	119.9
C9—C8—C7	103.83 (8)	C26—C27—C22	121.38 (10)
C9A—C9—C10	129.05 (9)	C26—C27—H27	119.3
C9A—C9—C8	107.22 (8)	C22—C27—H27	119.3
C10—C9—C8	123.72 (9)	C29—C28—C33	119.05 (10)
C9—C9A—C9B	140.59 (10)	C29—C28—C23	121.78 (9)
C9—C9A—C6B	110.99 (9)	C33—C28—C23	119.08 (9)
C9B—C9A—C6B	108.13 (9)	C28—C29—C30	120.29 (10)
C1—C9B—C6A1	118.89 (10)	C28—C29—H29	119.9
C1—C9B—C9A	135.14 (10)	C30—C29—H29	119.9
C6A1—C9B—C9A	105.69 (9)	C31—C30—C29	120.02 (10)
C15—C10—C11	118.12 (9)	C31—C30—H30	120.0
C15—C10—C9	122.35 (9)	C29—C30—H30	120.0
C11—C10—C9	119.47 (9)	C32—C31—C30	119.75 (10)
C12—C11—C10	121.20 (10)	C32—C31—H31	120.1
C12—C11—H11	119.4	C30—C31—H31	120.1
C10—C11—H11	119.4	C33—C32—C31	120.32 (10)
C13—C12—C11	120.04 (11)	C33—C32—H32	119.8
C13—C12—H12	120.0	C31—C32—H32	119.8
C11—C12—H12	120.0	C32—C33—C28	120.52 (10)
C12—C13—C14	119.69 (10)	C32—C33—H33	119.7
C12—C13—H13	120.2	C28—C33—H33	119.7
C14—C13—H13	120.2		
			
C9B—C1—C2—C3	1.0 (2)	C6B—C9A—C9B—C6A1	1.46 (11)
C1—C2—C3—C3A	−1.6 (2)	C9A—C9—C10—C15	−149.29 (11)
C2—C3—C3A—C6A1	0.6 (2)	C8—C9—C10—C15	30.91 (15)
C2—C3—C3A—C4	−177.27 (14)	C9A—C9—C10—C11	33.63 (16)
C6A1—C3A—C4—C5	−1.47 (19)	C8—C9—C10—C11	−146.17 (10)
C3—C3A—C4—C5	176.43 (14)	C15—C10—C11—C12	−0.39 (16)
C3A—C4—C5—C6	−0.2 (2)	C9—C10—C11—C12	176.81 (10)
C4—C5—C6—C6A	0.82 (19)	C10—C11—C12—C13	0.16 (17)
C5—C6—C6A—C6A1	0.26 (16)	C11—C12—C13—C14	0.30 (17)
C5—C6—C6A—C6B	−175.27 (12)	C12—C13—C14—C15	−0.52 (17)
C6—C6A—C6B—C7	6.8 (2)	C13—C14—C15—C10	0.29 (17)
C6A1—C6A—C6B—C7	−169.06 (14)	C11—C10—C15—C14	0.17 (16)
C6—C6A—C6B—C9A	178.67 (12)	C9—C10—C15—C14	−176.95 (10)
C6A1—C6A—C6B—C9A	2.78 (11)	C6B—C7—C16—C17	146.37 (11)
C4—C3A—C6A1—C9B	179.06 (11)	C8—C7—C16—C17	−26.63 (15)
C3—C3A—C6A1—C9B	0.94 (18)	C6B—C7—C16—C21	−32.59 (16)
C4—C3A—C6A1—C6A	2.63 (18)	C8—C7—C16—C21	154.40 (10)
C3—C3A—C6A1—C6A	−175.49 (11)	C21—C16—C17—C18	2.52 (16)
C6—C6A—C6A1—C3A	−2.05 (16)	C7—C16—C17—C18	−176.48 (10)
C6B—C6A—C6A1—C3A	174.75 (11)	C16—C17—C18—C19	−0.89 (18)
C6—C6A—C6A1—C9B	−178.79 (10)	C17—C18—C19—C20	−0.83 (19)
C6B—C6A—C6A1—C9B	−1.99 (12)	C18—C19—C20—C21	0.85 (19)
C6A—C6B—C7—C16	−3.9 (2)	C19—C20—C21—C16	0.86 (18)
C9A—C6B—C7—C16	−175.60 (10)	C17—C16—C21—C20	−2.51 (16)
C6A—C6B—C7—C8	169.94 (13)	C7—C16—C21—C20	176.49 (10)
C9A—C6B—C7—C8	−1.76 (11)	O—C8—C22—C27	7.52 (12)
C6B—C7—C8—O	−109.97 (9)	C9—C8—C22—C27	−111.57 (10)
C16—C7—C8—O	64.36 (12)	C7—C8—C22—C27	128.89 (10)
C6B—C7—C8—C22	130.30 (9)	O—C8—C22—C23	−172.49 (8)
C16—C7—C8—C22	−55.38 (12)	C9—C8—C22—C23	68.42 (12)
C6B—C7—C8—C9	5.26 (10)	C7—C8—C22—C23	−51.12 (12)
C16—C7—C8—C9	179.59 (9)	C27—C22—C23—C24	0.19 (14)
O—C8—C9—C9A	109.16 (9)	C8—C22—C23—C24	−179.80 (9)
C22—C8—C9—C9A	−132.62 (9)	C27—C22—C23—C28	−177.52 (9)
C7—C8—C9—C9A	−7.08 (10)	C8—C22—C23—C28	2.49 (14)
O—C8—C9—C10	−71.01 (11)	C22—C23—C24—C25	0.17 (15)
C22—C8—C9—C10	47.21 (13)	C28—C23—C24—C25	178.01 (9)
C7—C8—C9—C10	172.76 (9)	C23—C24—C25—C26	−0.63 (16)
C10—C9—C9A—C9B	13.9 (2)	C24—C25—C26—C27	0.73 (16)
C8—C9—C9A—C9B	−166.31 (13)	C25—C26—C27—C22	−0.38 (16)
C10—C9—C9A—C6B	−173.44 (10)	C23—C22—C27—C26	−0.08 (15)
C8—C9—C9A—C6B	6.38 (11)	C8—C22—C27—C26	179.91 (9)
C7—C6B—C9A—C9	−3.06 (12)	C24—C23—C28—C29	102.27 (12)
C6A—C6B—C9A—C9	−177.79 (9)	C22—C23—C28—C29	−79.98 (13)
C7—C6B—C9A—C9B	172.06 (9)	C24—C23—C28—C33	−74.17 (12)
C6A—C6B—C9A—C9B	−2.67 (11)	C22—C23—C28—C33	103.59 (12)
C2—C1—C9B—C6A1	0.52 (17)	C33—C28—C29—C30	−2.35 (15)
C2—C1—C9B—C9A	173.55 (12)	C23—C28—C29—C30	−178.79 (10)
C3A—C6A1—C9B—C1	−1.53 (17)	C28—C29—C30—C31	0.54 (16)
C6A—C6A1—C9B—C1	175.23 (10)	C29—C30—C31—C32	1.38 (17)
C3A—C6A1—C9B—C9A	−176.42 (10)	C30—C31—C32—C33	−1.47 (17)
C6A—C6A1—C9B—C9A	0.34 (12)	C31—C32—C33—C28	−0.37 (17)
C9—C9A—C9B—C1	0.6 (2)	C29—C28—C33—C32	2.27 (16)
C6B—C9A—C9B—C1	−172.20 (12)	C23—C28—C33—C32	178.81 (10)
C9—C9A—C9B—C6A1	174.27 (13)		
